# Tailoring Rheumatoid Arthritis Treatment through a Sex and Gender Lens

**DOI:** 10.3390/jcm13010055

**Published:** 2023-12-21

**Authors:** Loreto Carmona, Elena Aurrecoechea, María Jesús García de Yébenes

**Affiliations:** 1 Instituto de Salud Musculoesquelética, 28045 Madrid, Spain; 2 Rheumatology Department, Hospital Sierrallana, Instituto de Investigación Valdecilla (IDIVAL), 39300 Torrelavega, Spain

**Keywords:** rheumatoid arthritis, sex, gender, personalized medicine

## Abstract

Rheumatoid arthritis (RA) occurs more frequently in women than in men, and the studies that have addressed clinical and prognostic differences between the sexes are scarce and have contradictory results and methodological problems. The present work aims to evaluate sex- and gender-related differences in the clinical expression and prognosis of RA as well as on the impact on psychosocial variables, coping behavior, and healthcare use and access. By identifying between sex differences and gender-related outcomes in RA, it may be possible to design tailored therapeutic strategies that consider the differences and unmet needs. Being that sex, together with age, is the most relevant biomarker and health determinant, a so-called personalized medicine approach to RA must include clear guidance on what to do in case of differences.

## 1. Introduction

Using ‘sex’ and ‘gender’ interchangeably is not ideal, although they pose close connections in biology and disease. Although it may be important to distinguish conceptually between sex and gender, the reality is that both concepts are intimately related, resulting in an interaction in which it is practically impossible to establish a clear line of separation between them. However, despite the difficulties in conceptualizing and operationalizing these concepts, the importance of including sex and gender in biomedical research has been stressed in recent years [1,2]. Sex is a biological reality, including genetics and hormones, that modifies disease pathophysiology, expression, and clinical effect and distribution of pharmaceuticals. Studying sex encompasses a focus on the impact of potential genetic, hormonal, anatomical, and physiological differences on health and disease. Gender can be defined as the behavioral, cultural, or psychological traits typically associated with one’s sex resulting in a gendered division of labor, and socialization patterns that may impact an individual’s well-being. Studying gender entails analyzing the role of gender identity, norms, relations, and institutional aspects as possible sources of health inequalities.

There is no universal standard for measuring gender, and some review studies have shown persistent inadequacies in the conceptual understanding and methodological operationalization of gender in the biomedical field [2]. Because gender is very context-specific and is exceedingly hard to quantify, gender has been used in place of sex, and typically operationalized as men/women, although solely biological phenomena are being studied [2,3]. Moreover, gender and sex are so entwined that it might be challenging to set them apart [4,5].

The sociocultural construction of gender influences the behavior of populations, clinicians, and patients through different mechanisms, such as discriminatory values, norms, and beliefs, differential exposure and disease susceptibility, and even possible bias in health systems and health research [5,6].

The lack of information on the effect of biological (sex) and sociocultural (gender) differences in health may be due to different factors. Prior to the 1980s, most medical research ignored women and females as subjects of inquiry except when investigating ‘women’s health issues’—that is, issues directly related to reproduction or disorders seen only or predominantly in women. In most instances, female bodies were assumed to operate in the same ways as male bodies, and findings from research conducted exclusively on men were often uncritically generalized to women [1]. On the other hand, the history of excluding females from clinical studies is reflected in the 1977 US Food and Drug Administration (FDA) guidelines advising that women of childbearing potential should be excluded from drug trials. These recommendations resulted in inadequate female representation in clinical trials for decades to protect pregnant women and their offspring [7]. Fortunately, a gender-based approach to medicine has emerged to recognize and analyze sex differences in anatomical, physiological, biological, and therapeutic aspects of disease, as well as to assess potential effects of gender roles on other aspects of disease [8].

### 1.1. Gender and Chronic Diseases

Sex and gender are strong risk factors for practically every disease through genetic, epigenetic, and hormonal effects influencing physiology, illness, and drug metabolism, and social constructs of gender affecting behaviors, health engagement, and, subsequently, health [6].

As to chronic or noncommunicable diseases (NCD), the Global Burden of Disease (GBD) study has revealed several gender disparities, both in terms of mortality and morbidity burden, in cardiovascular disease (CVD), cancer, diabetes, renal disease, asthma, autoimmune diseases, migraine, spondyloarthritis, multiple sclerosis, Alzheimer’s disease, epilepsy, stroke, autism, depression, anxiety, and others [9,10]. Achieving equitable improvements in NCD morbidity and death requires recognizing and resolving these disparities.

### 1.2. Gender and Rheumatoid Arthritis

Rheumatoid arthritis (RA) is a chronic inflammatory autoimmune disease characterized by symmetrical polyarthritis that leads to progressive joint destruction and disability, plus damage to other organs and tissues. As in the majority of autoimmune conditions, RA occurs more frequently in women than in men (around three to one) [11], and there might be some sex differences in symptom severity and disease course, as well as in the effect of treatment and survival [12].

Female and male immune systems are different as they are affected by the distribution of hormones, the presence of two X chromosomes versus only one, and a singular response to environmental factors [11]. Several genes on the X chromosome regulate innate and adaptative immune responses. In addition, sex hormones are immunoregulatory, participate in the secretion of cytokines and chemokines, interact with inflammatory mediators, and play an essential role in pathobiological differences [13,14]. The role of environmental factors, including those in the psychosociological sphere, is less clear, probably reflecting the complex interaction among genes, hormones, and environment in autoimmunity [11,13].

Women with RA show a higher disease activity than their counterparts and respond worse to synthetic and biological disease-modifying therapies [14]. Also, pregnancy or childbearing desire influences the strategy for RA management. Consequently, to enhance treat-to-target performance, there is an ongoing demand for a precise reference to sex and gender issues in RA research. A more thorough understanding of the potential variables affecting sexual dimorphism in RA susceptibility, presentation pattern, disease activity, and outcome may result in more individualized treatment plans that minimize the illness’s burden [8].

The objective of this review is to analyze the impact of sex and gender on different aspects of RA to propose a sex- and gender-tailored approach to the management of RA.

## 2. Susceptibility to RA

Autoimmune diseases like RA show gender differences in incidence and immune response [15]. Different mechanisms for sex differences in autoimmunity have been proposed.

### 2.1. Genetic

Numerous immune-related genes are encoded on the X chromosome, and their overexpression may have sex-dependent effects on the immunological response. Men have one maternal X and one paternal Y chromosome, whereas women have two X chromosomes. In the early stages of development, one X chromosome is randomly silenced in females to prevent the double dose of X chromosome-derived proteins. Nevertheless, some X-linked genes are overexpressed in females due to incomplete X chromosomal inactivation, which allows 15% of the genes to elude inactivation [11].

The immune response is regulated by genetic factors, which could also account for the variations in RA susceptibility across sexes. The MHC, in particular class II alleles with a “shared epitope” (SE), and other non-MHC loci that seem to be sex influenced determine the genetic risk for RA [16]. For example, there is a direct correlation between the number of DR4 alleles of HLA and the frequency of RA in females [17]. Single nucleotide polymorphisms (SNPs) in cell-mediated immune-response genes may affect women and men differently. At least partially, they may account for sex differences in susceptibility to RA [18]. Immunoglobulin Fc receptors connect cellular and humoral immune responses. Male–female variations in RA may also be influenced by the sex connection of some SNPs of the Fc receptor-like genes (FCRL) [19]. MicroRNAs, short noncoding RNA molecules, regulate various processes, including the immune response. Dysregulation of microRNAs has been associated with autoimmune diseases, such as RA. MicroRNAs influence the expression of multiple protein-encoding genes at the posttranscriptional level. Women may be more susceptible to RA due to variations in the expression of X-chromosome-linked microRNAs or the genotype of certain microRNAs [11,20,21]. IL-4 promotes RA development via cytokine–receptor interaction, Th1 and Th2 cell differentiation, Th17 cell differentiation, T-cell receptor signaling pathway, cancer pathways, and hematopoietic cell lineage; curiously, IL-4 gene expression is lower in women with RA than in men [22].

### 2.2. Hormonal

The higher frequency of RA among women, especially before menopause, and the effect of pregnancy and oral contraception suppressing RA point out hormonal and reproductive factors [23,24]. Sex hormones may impact the development, risk, and course of autoimmune illnesses and several aspects of immune-system function. While testosterone and progesterone naturally depress the immune system, estrogens, especially 17-β estradiol (E2) and prolactin, operate as enhancers of humoral immunity at least, upregulating the production of immunoglobulins and downregulating inflammatory responses [11]. The inverse correlation between RA severity and androgen levels could be a reason why RA is less severe in men [25].

### 2.3. Gut Microbiota

Both the immune system and the gut microbiota have an impact on innate and adaptive immunological responses. This interaction may have significant effects on the emergence of inflammatory diseases. Several research studies using animal models have demonstrated the role of gut bacteria in sex bias in autoimmune disorders [11].

### 2.4. Lifestyle

Different studies have demonstrated that smoking is the strongest environmental factor in RA development. Smoking may induce citrullination of peptide antigens, and shared epitope alleles interact with smoking in the triggering of anticitrulline immunity that may lead to ACPA-positive RA. The risk of RA associated with smoking is generally higher among men than among women [26,27].

Population-based studies have shown a biological effect modification of gender on the smoking–RA association, such that smoking is a strong risk factor for RA in men and not so strong in women. The immunologic cascade triggered by smoking and leading to RA is modulated differently in men and in women. This effect is probably due to hormonal differences in the modulation of the immune cascade activated by smoking, leading to rheumatoid factor (RF) production and clinical RA [28]. Genetic and immunological factors may also play a role in the association between tobacco and AR, especially in genetically predisposed individuals. Persistent inflammation due to oxidative stress, proinflammatory state, autoantibody production, and epigenetic effects might be implicated in the autoimmunity of RA. Some studies also provide evidence that clinical responses to anti-TNF drugs used to treat RA may be adversely affected by smoking, and smoking may be related to extra-articular manifestations in RA [29].

In the context of environmental and lifestyle factors, obesity as a potential risk factor for the development of RA has been an area of interest for many years. Epidemiologic studies have shown a complex interaction between RA and obesity. Obesity would contribute to the development of seronegative RA, especially in younger women, although others suggested a decreased risk of RA among men, and observed associations may also be affected by residual confounding [26]. Different biologic mechanisms have been proposed to explain this association. Because adipose tissue is proinflammatory, obesity may also be an environmental risk factor for RA, with sex-specific effects. One large population-based case-control study demonstrated an association between obesity and RA, primarily for men with early disease onset [30]. Adipose tissue is an active secretory organ producing numerous bioactive molecules that regulate carbohydrate and lipid metabolism, immune function, and inflammation. Obesity produces chronic inflammation, and adipose tissue secretes different proinflammatory and anti-inflammatory factors, including the adipokines leptin, adiponectin, resistin, and visfatin as well as cytokines and chemokines, like TNF-a, IL-6, and others. An additional mechanism is based on the relationship between obesity and sex hormones. Obese men and women have higher serum levels of estrogens and androgens. Estrogen not only stimulates antibody (and autoantibody) production but also has a role in the breakdown of B-cell tolerance [31]. Indeed, obesity may also be associated with more severe or more refractory inflammation through increased levels of the inflammatory adipocytokines or decreased levels of the anti-inflammatory adipocytokine adiponectin [26].

Lastly, dysregulations of neuroendocrine-immune networks may underlie the gender-specific link that has also been observed between the onset of RA and childhood trauma, especially in women. Large prospective studies are necessary to elucidate the association between early-life stress and the risk for RA in genetically vulnerable individuals [32].

Despite a lack of consensus on how sexual dimorphism may affect the pattern and burden of disease, gender may indirectly increase RA susceptibility by influencing environmental and behavioral factors, which are known to be implicated in seropositive RA pathogenesis, in addition to the direct pathogenic effect of sex hormones on the immune system [8].

## 3. Disease Expression in Men and Women

The effect of the HLA-DRB1 shared epitope on RA susceptibility differs between men and women, thus raising the possibility of modifying disease expression by sex. Globally, the RA phenotype seems to be more severe in women than men, and women develop anti-CCP antibodies more frequently than men [33]. Women are also known to generally report more symptoms and poorer scores on most questionnaires, potentially affecting disease activity measures, treatment, or response to treatment between men and women [8,34,35].

The QUEST-RA study, a large multinational cohort, also showed gender differences in disease characteristics, including nodules (more common in men) and erosive disease (more prevalent in women) [34]. A study in Ecuador (*n* = 100) observed significant clinical differences, with men showing less disease activity—by physician’s assessment, painful and swollen joint counts, ESR, DAS28—than women, and lower functional and severe disability by HAQ-DI [25]. In a cross-sectional population-based study of 1128 RA patients from Latin America and Caribe (LAC), RA women showed a younger age at onset, longer disease duration, and higher prevalence of poly-autoimmunity and abdominal obesity. They were more likely to perform more household activities than men, whereas extra-articular manifestations were more frequent in men without differences in time to treatment. Some of these characteristics could explain the high rates of disability and worse prognosis observed in women with RA in these regions.

There are, however, some inconsistencies across studies. A retrospective study comparing the pattern of disease involvement in 438 Greek patients with early RA confirmed that women are affected more frequently and at a younger age than men. However, apart from a higher ESR, there were no significant differences in other clinical and laboratory parameters [36]. These results are similar to those obtained in the Spanish GENIRA study with 70 men and 70 women, in which no significant differences in disease activity—measured either by joint counts, physician or patient global scores, inflammatory markers, such as ESR or CRP, or by composite indices, such as DAS28- were observed [37].

Although differences can be attributed to biological factors, they could also be due to social factors related to gender rather than sex. The complexity of gender differences in health extends beyond notions of either physical or social disadvantages [38].

## 4. Prognosis and Mortality

Different outcome studies have shown gender differences in the disease course and prognosis of RA. Women seem to have a more aggressive early disease than men [39], and longitudinal studies have observed a more significant improvement in disease activity, function, and pain with time and treatment in men than in women [40]. The analysis of the long-term course of RA in the BARFOT (better antirheumatic pharmacotherapy) RA-inception cohort showed that being a woman was an independent predictor of persistent disease, defined as the absence of remission (DAS-28 < 2.6). However, this difference was unclear because most disease-related variables did not differ between women and men at the follow-up visits, indicating no gender bias in the measures of disease activity [41]. Female sex has also been described as the most powerful predictor of disability and data from early RA cohorts, with adverse HAQ trajectories more frequently seen in females [42]. Evidence points to similar disease activity in the early stages of the disease, followed by a worse clinical course among women over time, with lower remission rates, and these differences appear more pronounced in early RA [43].

In addition to disease progression, sex also appears to have a differential effect on RA mortality, although the literature data are not always consistent. Some studies show higher mortality rates in younger males but also a rapid increase in female mortality with age; this might be explained by specific hormonal protection of younger women, whose benefits are lost with menopause [39]. Prospective inception cohorts have shown that male sex is an independent predictor of long-term mortality [44,45]. Similarly, an Australian study covering trends from 1980 to 2015 found an increase in mortality among male patients compared with females, despite adequate treatment options, probably concerning differences in the CV risk profile [46]. However, other authors could not detect differences in RA mortality by gender. In a comprehensive review of 25 articles, Anderson et al. did not observe a clear association between sex and mortality in RA [47].

## 5. Comorbidity

Comorbidities pose a significant challenge in rheumatic diseases because of their impact on management and outcome. Independently of medication (glucocorticoids) and traditional risk factors (smoking), chronically active inflammation also predisposes to the development of comorbidity. The population-based international COMORA study showed that the diseases most frequently associated with RA are CVD, depression, asthma, solid neoplasms, and chronic obstructive pulmonary disease [48]. Gender differences in comorbidity can be summarized as a higher frequency of diabetes mellitus, peptic ulcer, and CV and respiratory diseases in men, with no significant impact on RA-related disability and QoL, and depression and osteoporosis being more frequent in women [37].

### 5.1. Depression

Depression is threefold more common in RA than in the general population and has a clear correlation with pain, fatigue, physical disability, and, consequently, poor quality of life (QoL). Women with RA are more frequently diagnosed with depression than men with RA. In turn, depression is independently associated with worse outcomes, other comorbidities, and mortality [37].

### 5.2. Cardiovascular Disease

Cardiovascular diseases are one of the most frequent comorbidities in RA and the leading cause of death [45,46]. Inflammation is, in addition to traditional risk factors, responsible for the development of accelerated atherosclerosis and heart disease. Cross-sectional studies comparing the CV risk profile by gender in low-activity RA without a history of CV disease have shown a higher atherosclerotic burden in male RA patients versus females, reflected by more increased carotid artery intima thickness, risk of 10-year CV death, atherogenic index, and NT-proBNP levels. However, men also smoke more and have lower HDL cholesterol levels, so the effect of classical CV risk factors cannot be ruled out [49]. Another possibility could be the documented underestimation of CV risk in women with RA using algorithms designed for the general population, such as the SCORE [50]. The measurement of structural vascular disease may be a more reliable option. Flow-mediated diameter percent change of the brachial artery is considered the gold standard of endothelial function and the best estimate of CV events risk. A prospective study showed a safer cardiovascular profile in RA females with better endothelial function than men. This may be surprising considering that women tend to have a more aggressive course, but this could be due to the beneficial effect of estrogens on endothelial function [51].

### 5.3. Osteoporosis

Osteoporosis is frequent in RA with the ongoing inflammatory process, low body mass index, lack of exercise, chronic use of glucocorticoids, and menopausal status [37]. Premenopausal women with RA also have more frequent osteoporosis than age-matched controls, and the same stands for men with RA. Oelzner et al. evaluated the frequency of osteoporosis by densitometry in a group of 551 RA patients. The results confirmed significant differences in the frequency of osteoporosis between postmenopausal women (55.7%), men (50.5%), and premenopausal women (18%). On the other hand, the relevance of the risk factors for osteoporosis was different in postmenopausal women (older age, high cumulative glucocorticoid dose, low BMI, and long disease duration). In contrast, the risk factors in premenopausal women and men are low BMI and high cumulative glucocorticoid dose, confirming the dependence of risk factors on gender and menopausal state [52].

### 5.4. Periodontitis

Periodontal disease is more prevalent in patients with active RA than in healthy individuals, and its severity is also associated with the severity of RA. The study of periodontal disease, using self-completed questionnaires, in 5600 Japanese RA patients in the IORRA cohort showed that 18.3% of patients reported a recent diagnosis of periodontitis, and 20.4% had a history of periodontitis. As in other comorbidities, the risk of periodontal disease also shows differences by sex, with women showing a higher risk, especially if aged and smokers [53].

## 6. Objective and Subjective Measures

Many indices are available to monitor RA, such as the DAS28 (with ESR or CRP), the Clinical Disease Activity Index (CDAI), or the Simplified Disease Activity Index (SDAI). They are all potentially influenced by sex and age, plus other factors such as body mass index (BMI) [54]. In general, women score more poorly than men, probably owing to higher “normal” levels of ESR and higher counts of tender joints (TJC) [34]. Women have higher mean ESR levels than men due to persistent ferropenic anemia hormonal factors. In older patients, the hormonal influences specific to women disappear, and older women are then affected by the same factors as older men [45]. On the other hand, sex differences in DAS28 may lead to a bias in assessing disease activity and response to treatment [55].

Nishino et al. assessed the effect of sex on composite measures in a large study [54]. They showed that sex differences in the composite measurements (DAS28-ESR, DAS28-CRP, CDAI, and SDAI) are only observed in remission based on DAS28-ESR, and this effect is mainly due to sex-related variability on ESR [56]. The score of the composite measures might be comparable between sexes, but not so the components; DAS28-CRP and SDAI may yield similar average scores despite a higher TJC28 and a lower CRP in women than in men. They suggest that almost 12% of men with RA could mistakenly be classified as in remission based on DAS28-ESR [54].

In addition to the sex effect on DAS28-ESR due to male–female differences in ESR, significantly higher SDAI and CDAI values have also been observed in women, probably because of a higher TJC and patient global assessment (PtGA) [57]. Both TJC and PtGA are affected by pain. Even though the mechanisms behind gender differences in pain perception are not fully clear, females are more sensitive to pain and report more daily pain than males [12,58,59]. A biopsychosocial perspective can explain these variations. Biologically, women may be more or less sensitive to pain than men. Women’s hormonal status may condition the processing of nociceptive stimuli by the central nervous system. Depression and other psychological variables, such as pain sensitivity, can also affect how someone perceives pain. Lastly, variations in socialization and sociocultural norms concerning the attention to and expression of pain may be the cause of gender disparities in pain perception [12,60]. Thus, gender differences may be due to the specific components of the indices, e.g., pain, and not to different degrees of inflammation [54,57,60,61]. Interestingly, differences in activity may not be accompanied by changes in the presence of radiographic erosions [57].

Both versions of DAS are used interchangeably to assess disease activity, treatment response, and treat-to-target approaches. However, the effect of sex on ESR may lead to discordance between the DAS28-CRP and DAS28-ESR. Discrepancies are more pronounced in older patients, women, and those at lower disease activity levels. The consequences of these effects can be significant, such as the availability of biological treatment or precluding the comparison of studies that have adopted different versions of DAS28. To avoid these limitations, a gender-stratified adjustment of the DAS28-CRP has been proposed to improve interscore agreement with DAS28-ESR. This adjustment allows us to consider observed biological differences in ESR levels between males and females [56].

The QUEST-RA study also showed sex differences in the relationship between DAS28 and body mass index (BMI). DAS28 scores increased with BMI only in women, and high BMI was associated with increased disease activity in RA [34,62]. Gender differences in the relationship between BMI and activity have also been observed using RAPID3 (routine assessment of patient index data) as the measure of activity. In a cross-sectional study of 451 RA patients, only female sex was found to have disease activity significantly associated with increasing BMI. The mean RAPID3 score values for each BMI category were statistically higher for females versus men [63]. The variation in the average amount of fat that a man and a female can have with the same BMI may cause sex variances in the association between BMI and disease activity. Males typically contribute more muscle mass to their BMI than females, who, in general, have a higher proportion of fat and a lower proportion of muscle mass. This could account for a rise in the proinflammatory state that causes disease activity to be higher in females than males [63].

Common depressive symptoms are more frequent in women, as already mentioned, and may affect all disease-activity metric scores (pain, global assessment and function, physician global assessment, and tender points), except the DAS, joint swelling, and serum biomarkers [37]. Finally, the higher HAQ score observed in women, even adjusting for disease severity, could be related to the underscoring of disability or greater muscle strength in men or a higher impact of pain on HAQ scores in women [61], men overestimating their functional capacity, or women having higher pain scores [59].

Table 1 shows the main objective and subjective differences in the indices and their components. 

## 7. Effect of Drugs

Empirical evidence points to female–male differences in biological treatment outcomes, which are probably multifactorial. Sex may influence effectiveness and safety through the effect of hormones on immune function, differences in drug pharmacokinetics and pharmacodynamics, and outcome measures [7,12]. In addition to sex, gender-related factors may also play a role in the effect of drugs. Understanding biological responses to drugs, especially adverse effects, in women relative to men has been negatively impacted by the underrepresentation of women in clinical trials. Also, social norms have caused disparities in health-seeking behavior and reporting, e.g., women report and experience more unfavorable effects than men due to higher perceived burdens, such as hair loss [7,64,65].

Prospective early RA cohorts are the best design to determine whether differences between sexes are present in the early stages of the disease or if they appear later. In a prospective DMARD-naïve early RA cohort (*n* = 292), Jawaheer et al. showed comparable disease activity early in the disease, followed by a worse course among women over time. The rate of change in activity scores was significantly influenced by gender, even after adjusting by other covariables like ESR, pain, function, or global health. At the same time, an increase in inflammation markers (ESR and CRP) was observed among women after six months, when disease activity scores started to diverge between men and women. It is possible that women are not as responsive to anti-inflammatory medication as men, which might explain the short-lived amelioration in levels of inflammatory markers. This increase in inflammation markers could be related to increased TJC and patient global scores and physician global assessment, observed after six months, and eventually higher disease activity among women [43].

The same authors investigated sex differences in response to anti-TNF in early (≤2 years since diagnosis) versus established RA in patients from the DANBIO registry. The outcome measure was EULAR response at 48 months. Among patients who initiated therapy within two years of diagnosis, men achieved better and faster EULAR responses than women (an interaction effect between sex and time was present). This gender difference in treatment response was not seen in patients who initiated anti-TNF therapy more than two years after diagnosis, suggesting that disease duration at baseline may determine the sex differences in response to treatment [66].

The effect of gender on the response to rituximab (RTX) shows inconsistent evidence. The British Society for Rheumatology Biologics Register showed a lower response rate in women than in men after six months of RTX treatment [67]. On the contrary, the French Autoimmunity and Rituximab registry showed that, after 12 months, remission rates (DAS28-ESR < 2.6) were higher in men if they had failed previously with anti-TNF but higher in women if they were anti-TNF naïve, suggesting that sex is probably not the determinant of response, but previous anti-TNF exposure [68].

As to the effect of gender on the response to abatacept, this was studied in the French Orencia and Rheumatoid arthritis registry but failed to detect any difference in response or remission rates, or even time to achieve them, between men and women after adjustment by age, disease duration, seropositivity, current DMARDs, previous anti-TNF, corticoid use, and disease activity, although the DAS28-ESR, TJC, and PtGA remained higher in women [69].

In a registry of 1912 RA patients who started biologic therapy, Lesuis et al. studied disease characteristics at the time of biologic initiation. The results confirmed gender differences in ESR, PtGA, TJC, HAQ, DAS28-ESR, and DAS28-CRP. However, no significant differences were observed in the prescribed biological treatment or the need for concurrent therapy with steroids, nonsteroidal anti-inflammatory drugs, or conventional DMARDs, with data equal to those observed in the GENIRA study [37]. According to their results, gender imbalance occurs only in subjective measures, such as pain, functional status, and QoL. These results may imply that subjective measurements are somewhat disregarded during the therapeutic decision-making process, which may point to female patients receiving insufficient care [59].

A meta-analysis by Fang et al. evaluated the impact of sex on the clinical response to six biological products (*n* = 5874) and found no significant differences in the ACR20 response rate between men and women. Interestingly, the analysis of subcomponents showed high heterogeneity between studies. Unfortunately, this meta-analysis did not evaluate gender differences in the treatment responses by subgroups of early and established RA [70]. We could not identify any study regarding differences in response or biological effect to jakinibs by sex.

## 8. Life Impact

There is a relationship between physical health and psychological well-being, although this link is far from perfect. Some individuals can maintain a high QoL, whereas others become depressed, and over time, many individuals tend to adapt to their condition such that psychological well-being improves even though physical debilitation may remain. Gender can be a potentially significant moderator in this process. Understanding the mechanisms by which gender operates can allow differential interventions to help men and women with RA achieve optimal QoL.

The gendered process appears to influence the psychological well-being of RA patients. Women have more depressive symptoms, higher levels of negative affect, somatic complaints, more passive coping strategies, and less socialization than men. Differential socialization patterns, leading to passive coping behaviors, may explain the observed gender differences in depressive symptoms. Women may be more likely to respond to stressful events by focusing internally on symptoms and their consequences [3].

The patient’s perception of their disease directly impacts their behavior, treatment compliance, and outcome. The social process also affects gender differences in adherence to treatment and illness perception. In a cross-sectional study of 320 RA patients, nonadherence was significantly associated with stress, disease activity, functional measures, and deformity, and female gender was an independent predictor of nonadherent behavior and more negative illness perception [71].

Women with RA show more depression, higher levels of disability (HAQ), and poorer QoL (SF36) than men. This may not be explained by overt disease-related biological differences but rather by differences in the patient’s comorbidity profile. As already mentioned, depressed RA patients have poorer long-term outcomes, more comorbidities, and increased mortality rates. Emotional responses to a physical illness characterized by pain and weakness are understandable, and somatic symptoms of depression might be expected as part of RA [37,72]. Differences in emotional distress between men and women with RA are mainly explained by functional ability and pain, as well as the characteristics of their paid work, with no independent effect for sex. Consequently, among employed RA patients with high levels of functional disability, gender is not a risk factor for emotional distress [73].

## 9. Coping

In general, RA is perceived by the patient as a source of chronic stress due to the associated limitations and symptoms. Stress coping strategies refer to the cognitive and behavioral efforts a person develops with specific demands of oneself or the environment. The inability to cope with stress results in a breakdown in health because of the depletion of the body’s hormonal and immune resources. The right way to cope with stress is of particular importance in chronically ill patients, and incompetent coping can contribute to the lack of effectiveness of the therapy [74,75].

The International Classification Functioning (ICF) emphasizes that contextual factors, which include both personal (gender) and environmental factors (healthcare system and attitudes of others), influence daily functioning. The perspectives of patients with RA, in terms of the impact of their chronic disease on everyday activities, are probably different. In general, societal expectations of women’s occupations and daily activities differ from those of men. Everyday activities mediate personal meaning and reflect one’s performance capacities, but continuing to perform these activities might be challenged due to the impact of RA. However, it may also depend on contextual factors, such as gender, individual and societal attitudes, health and social care systems, and policies. Masculinity is associated with competitiveness, self-control, strength, body performance, and productivity, whereas the caring role is more assigned to women. Men with a disability are more likely than women to fail to meet these social and cultural expectations and put first their paid work commitments over their health concerns. On the other hand, emotion regulation is a psychological determinant of health and is associated with psychological well-being, social and physical functioning, and disease severity. Compared to men, women with RA have a higher emotional orientation and reported stronger relationships between emotion regulation and the affective dimension of perceived health [76,77].

Gender differences were not found in the qualitative study of the situation-specific methods used to manage participation restrictions resulting from RA. However, women tend to offer more varied descriptions of their problems, especially in the domains of the ICF of domestic life and self-care. In contrast, men seldom report participation restrictions concerning domestic work, and these results could be explained, at least partly, by the existence of traditional gender roles. However, equity is developing when comparing most reported activities in which situation-specific coping was used by both women and men, namely, in remunerative employment and recreation and leisure [78].

In RA patients, depressive symptoms increase over time and with increased levels of pain, functional disability, and household work disability. Social status can contribute to depressive symptoms in different ways, including a sense of control, the ability to maintain the expectations of core social roles, and the ability to garner coping resources in the face of stressors. These findings illustrate the ongoing significance of social inequality for individuals with RA and offer additional confirmation of the necessity of comprehending and addressing variations in people’s capacities for coping with RA-related stressors [79].

## 10. Intersections of Gender

Studies on gender differences in pathological processes are complex due to the difficulty of measuring gender. The difficulty is even more significant in cases of sex–gender intersection. Hormone-replacement therapy, medication, and surgery can alter a transgender person’s hormonal status, which can, in turn, impact negatively on their health, e.g., increasing their risk of CV events [7].

Transgender and gender-diverse individuals (TGGD) have a gender identity that differs from their assigned sex at birth. They may affirm this identity through lifestyle modifications, gender-affirming hormone therapy (GAHT), or gender-affirmation surgery (GAS). There is not enough information on the epidemiology, pathophysiology, and clinical course of rheumatic diseases in transgender individuals. In 2022, Mathias et al. published an article with retrospective data on TGGD and a literature review of this population. In a retrospective analysis of 1053 patients seen in a rheumatology department over two years, seven TGGD patients (one RA) with rheumatic diseases were identified. The literature review found 11 studies with a total of 13 transgender patients (one RA).

The effect of GAHT on rheumatic disease possibly differs between estrogens and androgens, as most patients on exogenous testosterone experienced either no disease changes or improvement in disease activity. In contrast, most patients on exogenous estrogen experienced a possible acceleration of disease activity. The effect may also differ among different autoimmune diseases. The existence of a direct causation between the initiation of GAHT and the development or worsening of arthritis should be taken with caution due to the study’s limitations, mainly related to multiple confounders and probable publication bias. Higher-powered prospective studies are needed, and a registry would be valuable [80].

## 11. Gender and Health Services

Despite the importance of health care in patients with RA, little attention has been paid to whether there is differential access to or use of health care between men and women. Looking at differential access to health care by gender is not straightforward. Different factors are related, such as intrinsic patterns of healthcare use in men and women, socioeconomic barriers [77], and attitudes and behaviors [12].

The pattern of health care in RA patients is multifactorial and mainly explained by need-related factors, which supports the principle of equity. However, some gender differences have been observed. Women’s sex is an independent determinant of overall care. It increases the probability of receiving allied health and home care after adjusting for other characteristics, such as disease activity, duration, comorbidity, and functional status [81]. Concerning patient empowerment, younger and more educated women show a greater need for information and involvement in treatment decisions [82,83].

A delay in referral to subspecialty care puts patients at risk for delayed treatment and, thus, potentially worse outcomes. A retrospective analysis of a population-based cohort of incident RA has not shown differences between males and females in median time from first joint swelling to fulfillment of ACR/EULAR classification criteria, without gender impact on the time to the first DMARD therapy. However, among seronegative patients, there was a delay in meeting the 2010 criteria for females compared with males, with a longer time to start corticosteroid therapy in women. In patients with early seronegative disease, symptoms in females could be more often attributed to fibromyalgia, other noninflammatory conditions, or other rheumatic diseases [84].

Some authors have suggested that gender differences in RA may be due to differences in treatment prescription between men and women. A retrospective analysis of RA patients has not shown gender differences in the medication of MTX, dose, route of administration, time from disease onset, and percentage of patients receiving suboptimal doses. Overall, the data indicate that gender does not influence MTX therapy assigned by treating rheumatologists [85].

The gender of the attending physician may also play an essential role in health-care delivery. Female patients are more likely to obtain formal health care, provide more psychosocial information during a consultation than male patients, and show more preference for female physicians. In contrast, female physicians pay more attention to the psychosocial aspects of the complaints and use more gender-specific communication strategies than male physicians [77].

While it is evident that a gender bias characterizes RA reporting, perhaps there is also a gap in the evaluation of disease activity related to the different genders of the rheumatologist. In the analysis of 154 patients and their physicians, Duca et al. showed that subjective measures of global health status (GH) and disease activity are generally higher when collected by a female examiner. Female examiners recorded a more significant disease activity and a worse health status in both genders, with both male and female patients scoring higher levels of disease activity when evaluated by the female examiner compared to the male one. This observation is primarily attributable to variations of the GH and PtGA scores reported by patients, according to the absence of significant differences in the physical examination (TJC and SJC) performed by the examiners. Considering the chronic course of the disease, the physician–patient relationship is central in managing RA patients. Female physicians tend to exhibit higher emotional intensity in the physician–patient relationship. In this context, the higher emotional involvement between female physicians and patients may justify the higher values of PROs reported to the female examiner. Bearing in mind the impact of emotional well-being on the perceived disease activity, female physicians may better identify the subjective nature of complaints reported. This allows a more objective assessment of the global disease activity, especially in female patients [86].

## 12. Conclusions

A review of the impact of sex and gender on RA is presented. Although it is difficult to establish a clear line of separation between both concepts, sex seems to be more related to susceptibility, comorbidity (cardiovascular, osteoporosis, and periodontal disease), and objective measures (ESR, TJC, and BMI). In contrast, the influence of gender could be greater on environmental and behavioral risk factors, depression, subjective measures (pain, PtGA, HAQ, and QoL), life impact, and use of health services. Finally, it could be a mixed effect (sex and gender) on disease expression and the effect of drugs.

Gender medicine is a new paradigm focused on the differences between men and women in health and disease. RA might be triggered by a complex interaction between genetic, hormonal, environmental, and behavioral factors, all of which may be affected by sex. Comorbidities, reproductive issues, and measurement of disease activity all might affect treatment choices. Without a sex- and gender-sensitive and equitable approach to the management of RA, disparities in outcomes will persist.

Implementing sex and gender differences in scientific reports might be essential to equality and inclusivity. There is a critical need for research that addresses the biological (i.e., sex) as well as sociocultural (i.e., gender) causes of male–female disparities in immunotherapy responses, toxicities, and outcomes. Studies are also needed to define the influences of both patient and physician gender and their mutual interaction on the management of patients with RA.

Accounting for sex- and gender-related factors on health is an important challenge in research. The definition of research questions, experimental models, and statistical analysis should incorporate the complex, dynamic, and context-dependent constructs of sex and gender.

## Figures and Tables

**Table 1:** Differences in indices and their components by sex and gender.

Objective Measures: Differences by Sex	Subjective Measures: Differences by Gender
DAS-28	PtGA
CDAI	Pain
SDAI	Depression
RAPID-3	HAQ
ESR	Quality of life
TJC	
BMI	

## Data Availability

Not applicable.
